# Smoking cessation or reduction with nicotine replacement therapy: a placebo-controlled double blind trial with nicotine gum and inhaler

**DOI:** 10.1186/1471-2458-9-433

**Published:** 2009-11-27

**Authors:** Eva Kralikova, Jiri T Kozak, Thomas Rasmussen, Gunnar Gustavsson, Jacques Le Houezec

**Affiliations:** 1 Institute of Hygiene and Epidemiology, First Faculty of Medicine and the General University Hospital in PragueTobacco Dependence Treatment Centre of the 3rd Medical Department - Department of Endocrinology and Metabolism, First Faculty of Medicine, Charles University in Prague and the General University Hospital, Studnickova 7, 128 00 Prague 2, Czech Republic; 2 Hospital Kutna Hora, Vojtesska 26, 284 01 Kutna Hora, Czech Republic; 3 McNeil AB, Medical Affairs & Clinical Research, McNeil AB, PO Box 941, 251 09 Helsingborg, Sweden; 4 Consultant in Public Health, Rennes, France, and Special lecturer, University of Nottingham, UK. Amzer-Glas, 176 rue de Brest, 35000 Rennes, France

## Abstract

**Background:**

Even with effective smoking cessation medications, many smokers are unable to abruptly stop using tobacco. This finding has increased interest in smoking reduction as an interim step towards complete cessation.

**Methods:**

This multi-center, double-blind placebo-controlled study evaluated the efficacy and safety of nicotine 4 mg gum or nicotine 10 mg inhaler in helping smokers (N = 314) to reduce or quit smoking. It included smokers willing to control their smoking, and participants could set individual goals, to reduce or quit. The study was placebo-controlled, randomized in a ratio of 2:1 (Active:Placebo), and subjects could choose inhaler or gum after randomization. Outcome was short-term (from Week 6 to Month 4) and long-term (from Month 6 to Month 12) abstinence or reduction. Abstinence was defined as not a single cigarette smoked and expired CO readings of <10 ppm. Smoking reduction was defined as a reduction in number of cigarettes per day by 50% or more versus baseline, verified by a lower-than-baseline CO reading at each visit during the same periods.

**Results:**

Significantly more smokers managed to quit in the Active group than in the Placebo group. Sustained abstinence rates at 4 months were 42/209 (20.1%) subjects in the Active group and 9/105 (8.6%) subjects in the Placebo group (*p *= 0.009). Sustained abstinence rates at 12 months were 39/209 (18.7%) and 9/105 (8.6%), respectively (*p *= 0.019). Smoking reduction did not differ between the groups, either at short-term or long-term. Twelve-month reduction results were 17.2% vs. 18.1%, respectively. No serious adverse events were reported.

**Conclusion:**

In conclusion, treatment with 10 mg nicotine inhaler or 4 mg nicotine chewing gum resulted in a significantly higher abstinence rate than placebo. In addition a large number of smokers managed to reduce their cigarette consumption by more than 50% compared to baseline.

## Background

The efficacy of nicotine replacement therapy (NRT) as an aid to smoking cessation is well documented [1]. Because of its efficacy and safety profile, NRT is recommended as first-line pharmacological treatment for tobacco dependence. However, although most smokers state that they would like to quit, many are unable to abruptly stop using tobacco [2]. Some smokers have tried to quit and failed, so interventions that focus only on abrupt cessation are unlikely to motivate such smokers to alter their behavior.

Nicotine replacement therapy could also help smokers who are not able or willing to abruptly quit to reduce their smoking, by replacing some of the nicotine normally obtained from cigarettes [3]. Smoking reduction could represent a first step towards cessation, since in reducing their cigarette consumption smokers experience a degree of success in controlling their smoking behavior and this can encourage subsequent quit attempts [2,4]. Several previous studies of NRT for smoking reduction in smokers unable or not ready to stop resulted in about 10% of subjects being smoke-free at 12-24 months [5-8].

The present study investigated the efficacy of NRT to facilitate either smoking cessation or a reduction in smoking by 50% or more during a 6-month treatment period, with follow-up at 9 and 12 months. Our study differed from previous trials because smokers could choose one of two NRT products, gum or inhaler, and were given the opportunity to quit or reduce. The safety of NRT use, while smoking, was also investigated.

## Methods

### Study design

**Figure 1:**
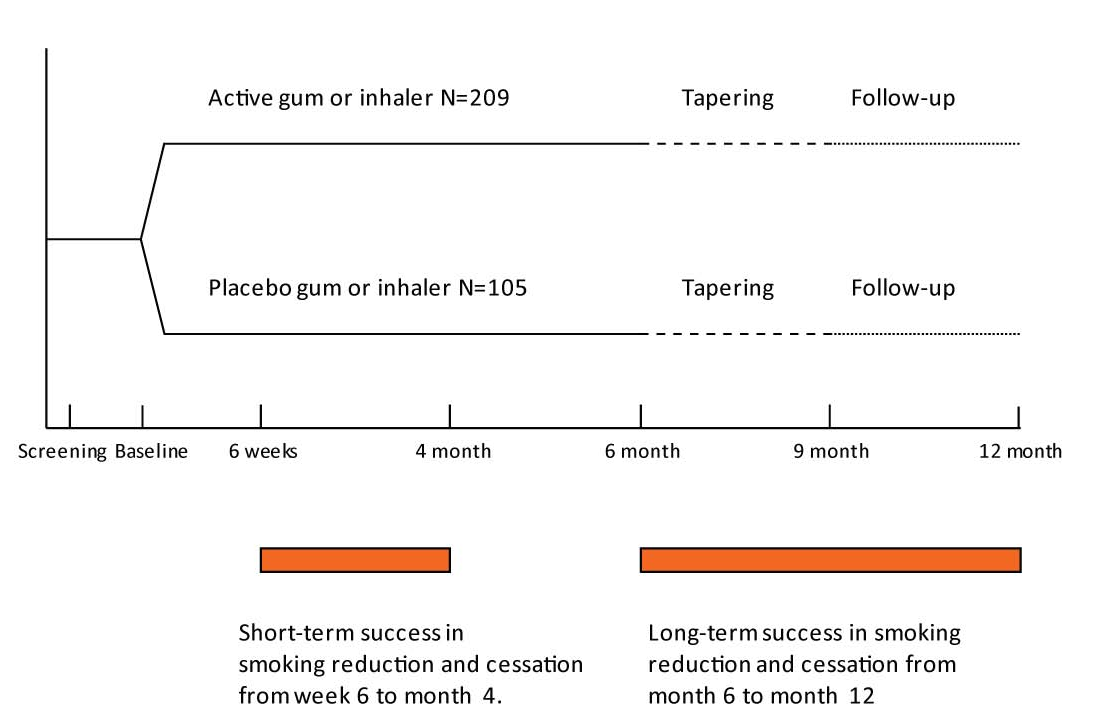
**Study design and success definitions**.

This was a double-blind, placebo-controlled trial with parallel groups, performed at two medical centers (Prague and Kutná Hora) in the Czech Republic. Two NRT products were used, nicotine 10 mg inhaler and nicotine 4 mg gum. The choice of NRT was offered to optimize compliance with treatment to achieve the best possible quit rate or reduction rate. Smoking cessation was recommended but not mandatory; subjects who could not achieve or maintain abstinence were asked to reduce their smoking as much as possible. The study design is shown in Figure 1.

### Subjects

Subjects were recruited via an advertisement in the local Prague newspaper "Metro" (distributed free on Prague public transport), and using local leaflets in Kutna Hora. The ad and leaflet had a standardized content that invited smokers to participate in a clinical trial to control their smoking. Both the newspaper ads and the subject information put equal emphasis on cessation and reduction. Respondents were initially screened by telephone; up to 325 eligible smokers were invited to participate in the study and to come to the personal screening. Eligible subjects were at least 18 years old, smoked ≥ 15 cigarettes per day (cpd), had smoked regularly for ≥ 3 years, had an expired air carbon monoxide (CO) level of ≥ 10 parts per million (ppm), wanted to reduce their smoking, and had previously made at least one failed quit attempt (i.e., all participants had experienced difficulties trying to stop on their own), but did not have to be motivated to quit smoking. All participants provided informed consent, and the ethics committee of the First Faculty of Medicine, and the General University Hospital, Charles University, Prague, approved the study. Eligible subjects received brief smoking cessation/reduction support and information about the use of NRT.

Exclusion criteria included current use of NRT or other nicotine-containing products (such as cigars, pipes, snuff), and current involvement in other smoking cessation or smoking reduction programs. Unstable angina pectoris or myocardial infarction within the previous 3 months, pregnancy/lactation or intended pregnancy, psychiatric treatment or medication, and co-existing alcohol or other drug problems precluded participation.

### Procedure

At inclusion and during the study, subjects received brief (up to 10 minutes) behavioral smoking reduction/cessation support. Subjects were instructed to reduce their smoking by replacing as many cigarettes as possible with treatment, either inhaler (nicotine 10 mg or placebo) or gum (nicotine 4 mg or placebo), according to their choice. A randomization ratio of 2:1 (Active:Placebo) was used to accommodate the need for a control group while, for ethical reasons, allowing most of the population use active medication. The product selected at randomization (gum or inhaler) could not later be changed. Commercially available nicotine inhaler and nicotine gum (Nicorette^®^, McNeil AB) were used as the active investigational medications. The placebo groups received matching treatment that did not contain nicotine.

The treatment schedule was 6 months of full treatment, followed by up to 3 months of voluntary tapering to prevent relapse to smoking. Follow-up visits were at 9 and 12 months. The recommended doses were 6-12 cartridges daily, but not more than 12 cartridges in any 24-hour period, for the inhaler, and *ad libitum *use, up to a maximum of 24 pieces/day, for the gum. Treatment compliance was measured at each visit; subjects reported the quantity of used medication and returned any unused medication.

### Assessments

Nine clinic visits were scheduled: screening, baseline, Weeks 2, 6, 12, and Months 4, 6, 9 and 12. Demographic data, smoking history and nicotine dependence using the Fagerström Test for Nicotine Dependence (FTND) [9] was recorded at baseline. Expired CO levels were assessed at each visit. Intention to quit was measured at all visits from baseline onwards. Plasma cotinine was measured at baseline, Week 6, and Months 4, 6 and 12. Regarding safety, adverse events were assessed at all visits from Week 2 onwards. Cardiovascular biomarkers were also assessed, but the biomarker results are not presented in this paper. Smoking consumption was monitored from Week 2 to Month 12, and use and acceptability of study treatment were assessed from Week 2 to Month 6 using a standard questionnaire.

### Outcomes

The treatment goal was either complete abstinence or reduction of ≥ 50% in the number of cpd smoked. Sustained abstinence was defined as not a single cigarette smoked and expired CO readings <10 ppm at each visit. Point prevalence abstinence was defined as not a single cigarette smoked during the previous 7 days and a CO reading <10 ppm. Smoking reduction was defined as a reduction in smoking by 50% or more versus baseline, verified by a lower-than-baseline CO reading at each visit. As the subjects were free to make quit attempts at any time during treatment, the standard definition of sustained, complete abstinence from study start is therefore not adequate. Instead, sustained abstinence and reduction are reported for an early short-term period (from Week 6 to Month 4) and a later long-term period (from Month 6 to Month 12). Subjects who achieved sustained abstinence were termed Abstainers, and subjects who reduced smoking by ≥ 50% were termed Reducers.

### Statistical analysis

The primary analysis was an intention-to-treat analysis, which included all subjects who received treatment. Dropouts were regarded as treatment failures. All statistical tests were two-tailed and at a 5% significance level. No formal adjustments for multiplicity were made, but p-values were presented for each test to allow for relevant interpretation. The Wilcoxon rank sum test was used to test intra-individual differences from baseline to 4 and 12 months in each outcome group. Primary efficacy results were analyzed using Pearson's Chi-square test. In a separate analysis of efficacy using logistic regression, baseline FTND score, CO level and cigarette consumption were tested as prognostic factors.

Results from previous studies of cessation/reduction suggested success rates of reducing smoking of 50% in the Active treatment group and 30% in the Placebo group. Given these conditions, 140 + 70 = 210 subjects (ratio 2:1) were needed for an alpha of 0.05 and a power of 80%. A total of 325 subjects, well above the number needed, attended the screening visit.

## Results

### Study population

Out of the 325 eligible subjects, 314 smokers (131 males, 183 females), recruited between January and May 1999 entered the trial (11 enrolled subjects never attended baseline). Their mean age was 46 (range 20-68) years, mean baseline cigarette consumption was 25 cpd (range 13*-70), and mean age at onset of smoking was 18 years (range 8-42). Mean baseline value for expired CO was 23 ppm (range 1*-58) and the mean FTND score was 6.0 (range 0-10) (Table 1). All participants who fulfilled the inclusion criteria were randomised at screening visit, but 13 subjects had reduced smoking between the screening and baseline visits, which explains the low number of cpd, CO level and FTND score at baseline in some subjects.

**Table 1:** Demographic characteristics at baseline. Values are mean ± standard deviation (range)

Characteristic	Active NRT (n = 209)	Placebo (n = 105)
Gender	89 M/120 F	42 M/63 F
Age (years)	46.1 ± 10.5 (20-68)	46.6 ± 10.0 (20-67)
Age started smoking (years)	18.3 ± 3.3 (8-30)	18.6 ± 3.9 (12-42)
Cigarettes smoked per day	25.7 ± 9.8 (13*-60)	25.2 ± 8.2 (15-70)
Expired CO level (ppm)	22.9 ± 10.0 (1*-58)	23.9 ± 9.0 (9-54)
Total FTND score	5.8 ± 2.1 (0*-10)	6.2 ± 2.1 (1*-10)

Abbreviations: CO = carbon monoxide; ppm = parts per million, FTND = Fagerström Test of Nicotine Dependence

* All participants fulfilling inclusion criteria were randomised at screening visit, but 12 subjects had reduced smoking between screening and baseline visit explaining the low number of cpd, CO and FTND at baseline in some subjects.

### Treatment choice and compliance

Two hundred and nine subjects were allocated to Active NRT, and 105 to Placebo. After randomization subjects could choose between inhaler and gum within their own treatment code; 263 subjects chose inhaler (111 males, 152 females), and 51 chose gum (20 males, 31 females). The inhaler was chosen by 84% of subjects (85% males, 83% females).

In the Active group, 84% of the 196 subjects who attended the Week 2 visit reported daily use of treatment, compared to 80% of the 95 subjects in the Placebo group. Corresponding figures at 9 months were 58/130 (45%) and 24/62 (39%) in the Active and Placebo groups, respectively. Comparisons of compliance showed that gum users were more perseverant; 68% in the Active group and 67% in the Placebo group were still using the product daily at 9 months, vs. 41% and 34%, respectively, in the inhaler groups. The main reason for not using treatment was "did not need it" in both groups.

### Efficacy

**Figure 2:**
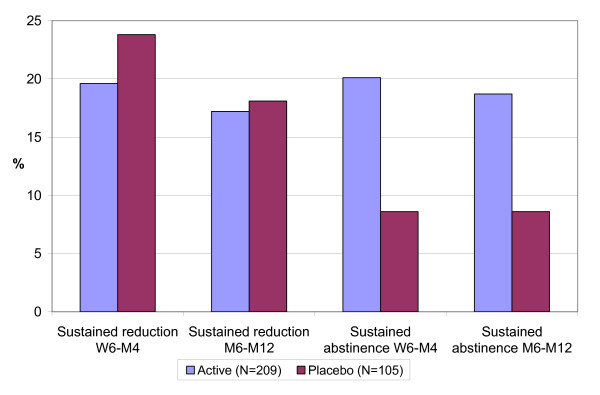
**Short title: Sustained reduction and abstinence from week 6 to month 4, and month 6-12**. Detailed legend: Sustained reduction and abstinence from week 6 to month 4, and from month 6 to month 12 in active vs. placebo groups (see text for definitions and *p *values).

Sustained and point prevalence abstinence rates were significantly higher in the Active group than the Placebo group, both at short-term (from Week 6 to Month 4) and long-term (from Month 6 to Month 12) follow-up (Table 2; Figure 2). Sustained abstinence rates at 4 months were 20.1% (Active) vs. 8.6% (Placebo; *p *= 0.009); 12-month results were 18.7% vs. 8.6%, respectively (*p *= 0.019). Point prevalence abstinence rates were Active 26.3% vs. Placebo 13.3% at 4 months (*p *= 0.009), and 21.5% vs. 10.5%, respectively, at 12 months (*p *= 0.016).

**Table 2:** Primary efficacy results: smoking cessation and smoking reduction.

Definition	Active (n = 209)		Placebo (n = 105)		χ^2 ^p-value
			
	n	%	n	%	
Sustained abstinence from W6 to M4	42	20.1	9	8.6	0.009
Sustained abstinence from M6 to M12	39	18.7	9	8.6	0.019
Point prevalence abstinence at 4 M	55	26.3	14	13.3	0.009
Point prevalence abstinence at 12 M	45	21.5	11	10.5	0.016
Sustained reduction from W6 to M4	41	19.6	25	23.8	
Sustained reduction from M6 to M12	36	17.2	19	18.1	

Summary of primary efficacy results: smoking cessation and smoking reduction from week 6 to month 4, and from month 6 to month 12.

Although a large proportion of subjects reduced their smoking by more than 50%, there was no statistically significant difference between Active and Placebo groups, either at short-term (Week 6 through Month 4) or long-term (Month 6 through Month 12) follow-up (Table 2; Figure 2). At 4 months, 19.6% of the Active group and 23.8% of the Placebo group had successfully reduced smoking. Twelve-month reduction results were 17.2% vs. 18.1%, respectively.

**Figure 3:**
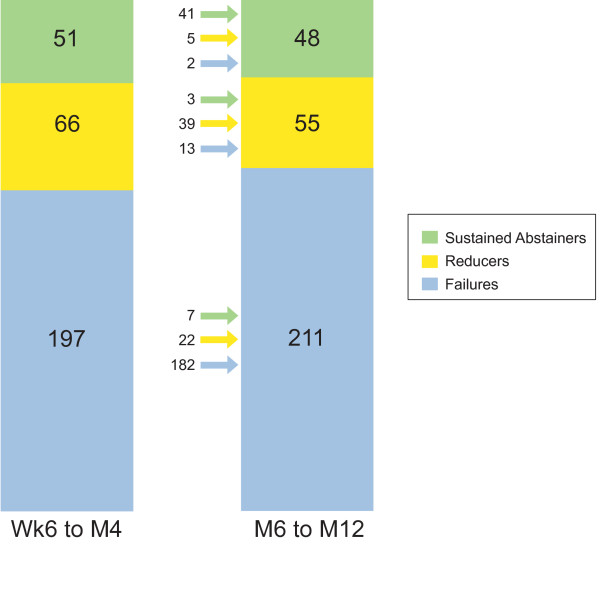
**Number of subjects in each outcome group between Week 6-Month 4 and Months 6-12**. Detailed legend: Number of subjects in each outcome group (abstainers, reducers, failures) between Week 6-Month 4 and Month 6-Month 12. The arrows to the left of the column for Month 6-Month 12 denote the previous status of these subjects during Week 6-Month 4.

Figure 3 shows the total number of abstainers, reducers and failures in the short term and long term. It illustrates how subjects migrated between outcome classifications; as these were two separate subgroups, subjects could go from being, for example, an abstainer in the first phase to a reducer in the second phase. Eighty percent (41/51) of the short-term abstainers were also long-term abstainers; 7.6% of short-term reducers and 1% of short-term failures became abstinent long term. 6.6% of initial failures succeeded in reducing long-term.

### Prognostic factors

Baseline FTND score, CO level and cigarette consumption were tested as prognostic factors. In this study, only expired CO was related to successful smoking reduction or cessation: a lower baseline CO was associated with higher success probability, chi-square *p *= 0.01 and *p *< 0.05 for 4-month and 12-month reduction or cessation, respectively.

### Intention to quit

Subjects who were still smoking were asked: "Do you intend to quit smoking completely in the next month?" Responses were rated from 0 = "definitely not" to 4 = "definitely".

At 4 months, neither the 52 Reducers nor 80 Failures who responded had changed their intention to quit. Reducers had a mean (SD) score of 2.9 (1.0) at baseline and 3.1 (1.1) at month 4. Failures had scores of 2.8 (1.2) and 2.7 (1.1), respectively. Long-term reducers (N = 52) decreased their mean score from 3.1 (0.9) at baseline to 2.3 (1.2) at Month 12 (Wilcoxon *p *< 0.001), compared to ratings of 2.7 (1.1) versus 2.5 (1.2) (NS) in 81 failures.

### Plasma cotinine levels

At 4 months, mean plasma cotinine concentration had significantly decreased versus baseline in Abstainers (Wilcoxon; *p *≤ 0.001) and Reducers (*p *=≤ 0.001), but was unchanged in Failures (Table 3). At 12 months, mean plasma cotinine was decreased only in Abstainers (*p *< 0.001).

**Table 3:** Plasma cotinine and expired carbon monoxide (CO) levels

	Cotinine (ng/ml)				CO (ppm)			
	
Outcome group	4 months (baseline)	*p*	12 months (baseline)	*p*	4 months (baseline)	*p*	12 months (baseline)	*p*
Abstainers*	80 (220) N = 46	<.001	40 (232) N = 45	<.001	4.5 (23.0) N = 51	<.001	2.7 (22.4) N = 48	<.001
Reducers*	184 (279) N = 63	<.001	216 (253) N = 50	NS	13.1 (26.4) N = 66	<.001	11.7 (26.3) N = 55	<.001
**Failures**	265 (259) N = 73	NS	271 (257) N = 80	NS	20.7 (19.4) N = 88	NS	17.2 (20.4) N = 90	<.001

*Includes all subjects who were classed as Abstainers and Reducers in both the Active and Placebo treatment groups.

### Expired carbon monoxide

At 4 months, mean expired CO had decreased versus baseline in Abstainers (Wilcoxon; *p *< 0.001), and Reducers (*p *< 0.001), but was unchanged in Failures (Table 3). At 12 months, mean expired CO had decreased in Abstainers (*p *< 0.001), Reducers (*p *< 0.001), and Failures (*p *< 0.001). Although the decrease in CO was statistically significant in all three groups, the magnitude of decrease was far greater in Abstainers and Reducers than in Failures.

### Adverse events

Concomitant use of 10 mg nicotine inhaler or 4 mg nicotine gum with cigarette smoking was well tolerated, and no unexpected adverse events occurred during the study. The incidence of adverse events was higher in the Active group (82 events in 209 subjects) than the Placebo group (26 events in 105 subjects). Of the 82 events in the Active group, 47 (57%) were mild, 28 (34%) moderate and 7 (9%) severe. Of the 26 events in the Placebo group, 22 (85%) were mild, 3 (12%) moderate and 1 (4%) severe. The most common events were throat/mouth irritation and cough. Signs of possible nicotine-related systemic events were reported in 6 subjects in the Active group (2 nausea, 3 vertigo, 1 palpitation) compared to 1 in the Placebo group (vertigo).

Treatment-related adverse events were cough (Active 9 vs. Placebo 3), heartburn (3 vs. 0), and mouth, throat and tongue irritation (15 vs. 1) with the inhaler, and mouth and throat irritation (Active 2 vs. Placebo 1), salivation (2 vs. 0), upset stomach and hiccups (3 vs. 0) with the gum.

## Discussion

The purpose of this study was to investigate whether smokers prepared to control their smoking, but not necessarily prepared to quit smoking, could reduce or quit with a choice of one of two NRT products, nicotine 4 mg gum or nicotine inhaler. The study also evaluated the safety of NRT used concomitantly with cigarette smoking.

In the short-term, between Week 6 and Month 4, Active treatment achieved a significantly higher rate of sustained abstinence than Placebo (*p *≤ 0.009). In the long-term (Months 6-12), sustained abstinence was significantly superior with Active treatment (*p *≤ 0.019). Subjects could choose between two NRT forms, although use of a preferred form has not been shown to increase outcome [10]. In our study the outcome did not differ between gum and inhaler users, although another study has suggested that adherence to inhaler can be quite low [11]. The subjects' preference for the inhaler (chosen by four-fifths of subjects) over the gum may have been due to the novelty of the inhaler, which was not available on the Czech market at the time this study was performed. However, gum users were more compliant than inhaler users, which may reflect the novelty of the inhaler; subjects chose the inhaler because it was new, but found it more difficult to use than gum. Reasons may be that gum is more discreet to use in public, and that the inhaler has to be used much more intensively than a cigarette to obtain an adequate dose of nicotine [10].

Overall, NRT increases long-term cessation rates by 50 to 70%, irrespective of the formulation, in smokers motivated to quit [1]. Classical smoking cessation trials enroll subjects motivated to quit, whereas our study recruited smokers who wanted to 'control their smoking', which implied either reducing cigarette consumption or immediate quitting. Nonetheless, we observed similar results: short- and long-term abstinence in the Active group was approximately double that in the Placebo group. The one-year abstinence rate in our study (18.7%) was higher than that observed in a number of previous trials of NRT products for smoking reduction (8-12%) [5-8]. This is not surprising, given the different study populations: our study invited smokers who wanted to control their smoking (in any way), whereas previous studies of NRT for smoking reduction specifically enrolled smokers unable or not motivated to quit [5-8]. However, the results from our study confirm earlier findings that offering smoking reduction does not undermine cessation [4].

The reduction rate was also high in the placebo group. If smokers could reduce, and sustain that reduction, active medications might help even more smokers to quit because they would be starting from a lower level of nicotine dependence, if they had already reduced smoking, either with or without the help of treatment. This hypothesis could be tested in future studies.

The study design included a screening visit 2 weeks before baseline. Some subjects reduced their cigarette consumption between the screening visit and baseline visit, with the result that baseline measurements of cigarette smoking, carbon monoxide and FTND did not accurately reflect smoking levels before the screening visit. This may have negatively affected the smoking reduction outcome in this study. Twelve of the subjects who fulfilled the inclusion criteria and were randomized at the screening visit had an expired CO level <10 ppm at the baseline visit, either because it was an early morning visit or because they had already reduced smoking. One subject indicated at baseline that he now smoked less than 15 cpd; at study end, this subject had failed to quit or reduce smoking. Another subject, who achieved abstinence (short- and long-term), had a baseline FTND score of zero but fulfilled the inclusion criteria for cpd and expired CO. The low baseline values of these subjects did not affect the cessation outcome, and excluding them from the analyses did not change the result, although their low baseline CO level made it difficult for these subjects to fulfill the criteria of reducers.

Previous studies that evaluated the effect of smoking reduction have recruited smokers who were not willing to immediately quit, and have also studied only one type of NRT [5-8]. This is different from a real-life situation where smokers are exposed to different smoking cessation strategies and NRT is available over-the-counter, and sometimes in self-selection areas of pharmacies or in general sales, where smokers can choose between different products. This study recruited smokers who were either willing to quit, or only to reduce their number of cigarettes smoked per day. They were also exposed to a choice of two different NRT products. This study is therefore more similar to the real life situation and verifies the efficacy of nicotine gum and inhaler for smoking cessation when using individual strategies to reach this goal.

In our trial, both the newspaper ads and subject information put equal emphasis on cessation and reduction. However, recent experience shows that most volunteers are usually more motivated to quit than to reduce smoking, unless the recruitment procedure is specifically adapted to enroll "reducers". In a comparable study, specific inclusion/exclusion criteria to differentiate motivation to reduce from motivation to quit were successfully applied [7].

Concurrent use of NRT and smoking is safe, as confirmed in the present study, and generally does not increase blood nicotine levels, particularly with nicotine gum or inhaler [12]. Nor are other biomarkers increased during smoking reduction while using nicotine inhaler [13]. The reduction in expired CO in reducers was highly significant, even at 12 months, which confirmed the efficacy of smoking reduction in our subjects.

Current smoking cessation interventions are aimed at smokers who are already motivated and preparing to quit. A broader range of interventions is needed in order to bring more smokers into treatment and increase the numbers who are motivated to make quit attempts. In addition to abrupt cessation, treatment guidelines should recommend NRT-assisted reduction, as a substantial body of evidence shows that gradually cutting down smoking can increase subsequent smoking cessation among smokers not currently interested in quitting [4-8,14,15].

## Conclusion

In conclusion, NRT (nicotine gum or inhaler) doubled the quit rate compared to placebo and was safe. Active NRT achieved sustained abstinence rates of 20.1% at 4 months and 18.7% at 12 months, which is similar to NRT findings in studies that enrolled only smokers motivated to quit. In addition, a large number of smokers managed to reduce their cigarette consumption by more than 50% compared to baseline.

## Competing interests

This study was funded by McNeil AB, Helsingborg, Sweden. McNeil AB manufactures a range of nicotine replacement products, including nicotine gum and nicotine inhaler. Eva Kralikova and Jiri Kozak† received funding from McNeil AB to perform this study (and have previously received payment from other pharmaceutical companies). Thomas Rasmussen and Gunnar Gustavsson are employees of McNeil AB. Jacques Le Houezec is a consultant in tobacco dependence for both the pharmaceutical industry and the public sector.

## Authors' contributions

EK, and JK† performed the study. TR performed the statistical analysis and participated in the writing of the manuscript. EK, GG and JLH wrote the manuscript. All authors read and approved the final manuscript.

## Pre-publication history

The pre-publication history for this paper can be accessed here:

http://www.biomedcentral.com/1471-2458/9/433/prepub
